# Supplementary material for: Non-lytic spread of poliovirus requires the nonstructural protein 3CD
Source: mBio. 2024 Dec 12;16(1):e03276-24. doi: 10.1128/mbio.03276-24 (PMC11708018; doi:10.1128/mbio.03276-24)
Supplement: Supplemental Material — Supplemental figures and legends for supplemental movies. [file mbio.03276-24-s0001.docx]

**
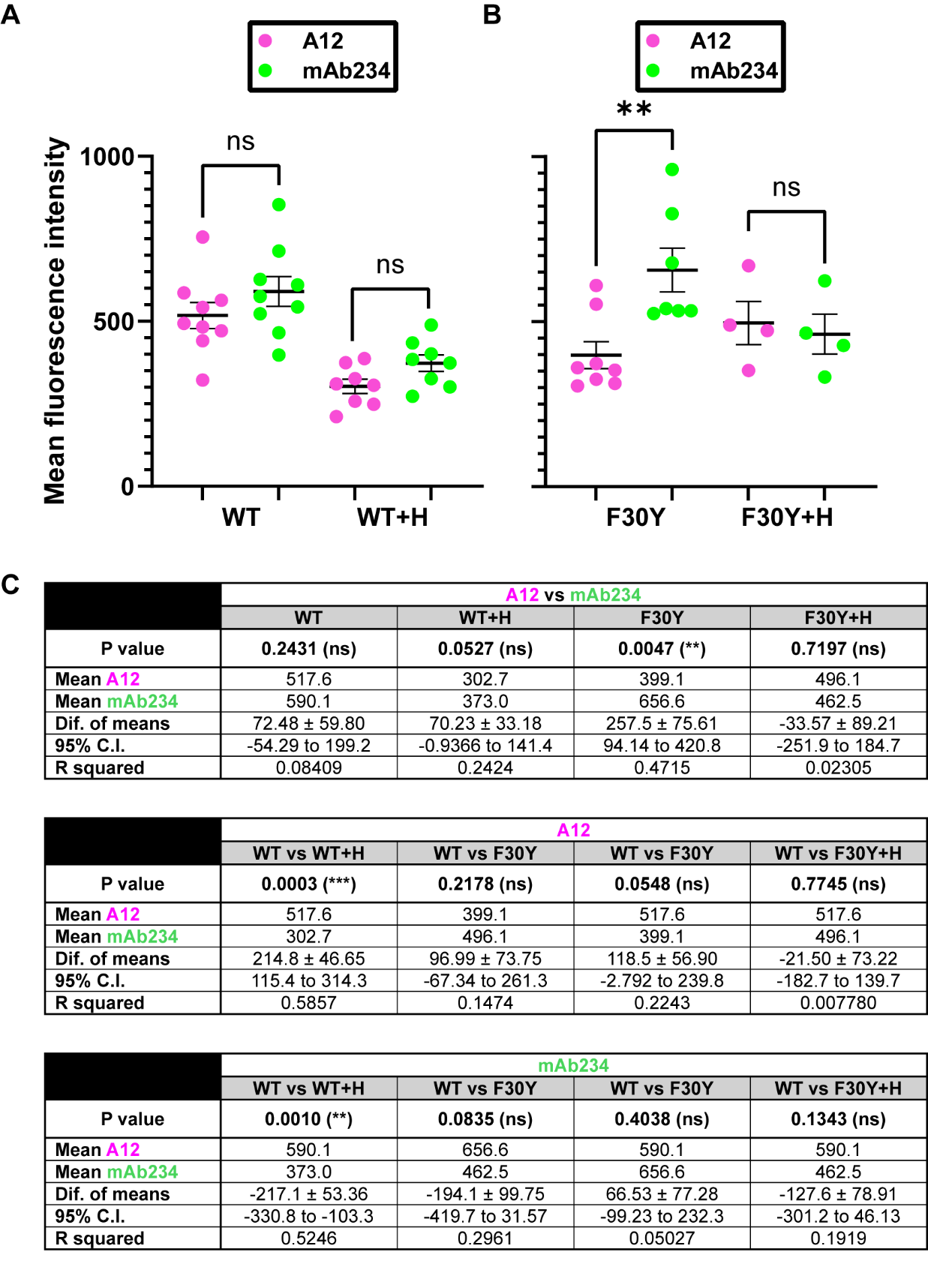
**

**Figure S1. A12 and mAb234 fluorescence intensity analysis of PV-infected cells**

**(A)** **Confocal immunofluorescence imaging intensity measurements of A12 and MAb234 in WT PV-infected HeLa cells.** Images illustrate intensity measurements of whole cells in representative immunofluorescence image fields of WT-infected HeLa cells (MOI of 10) in the presence and absence of hydantoin, as described in **Fig 2C**. Mean fluorescence intensity is plotted on the y-axis, and the conditions on the x-axis. **(B)** **Confocal immunofluorescence imaging intensity measurements of A12 and MAb234 in F30Y PV-infected HeLa cells.** Images illustrate intensity measurements of whole cells in representative immunofluorescence image fields of F30Y-infected HeLa cells (MOI of 10) in the presence and absence of hydantoin, as described in **Fig 2C**. Mean fluorescence intensity is plotted on the y-axis, and the conditions on the x-axis. **(C) Statistical analysis on fluorescence intensity measurements.** An unpaired student t-test analysis was performed to compare the intensity measurements of WT and F30Y-infected cells in the described conditions. A p-value lower than 0.005 was considered significant with a 95% confidence interval.

**
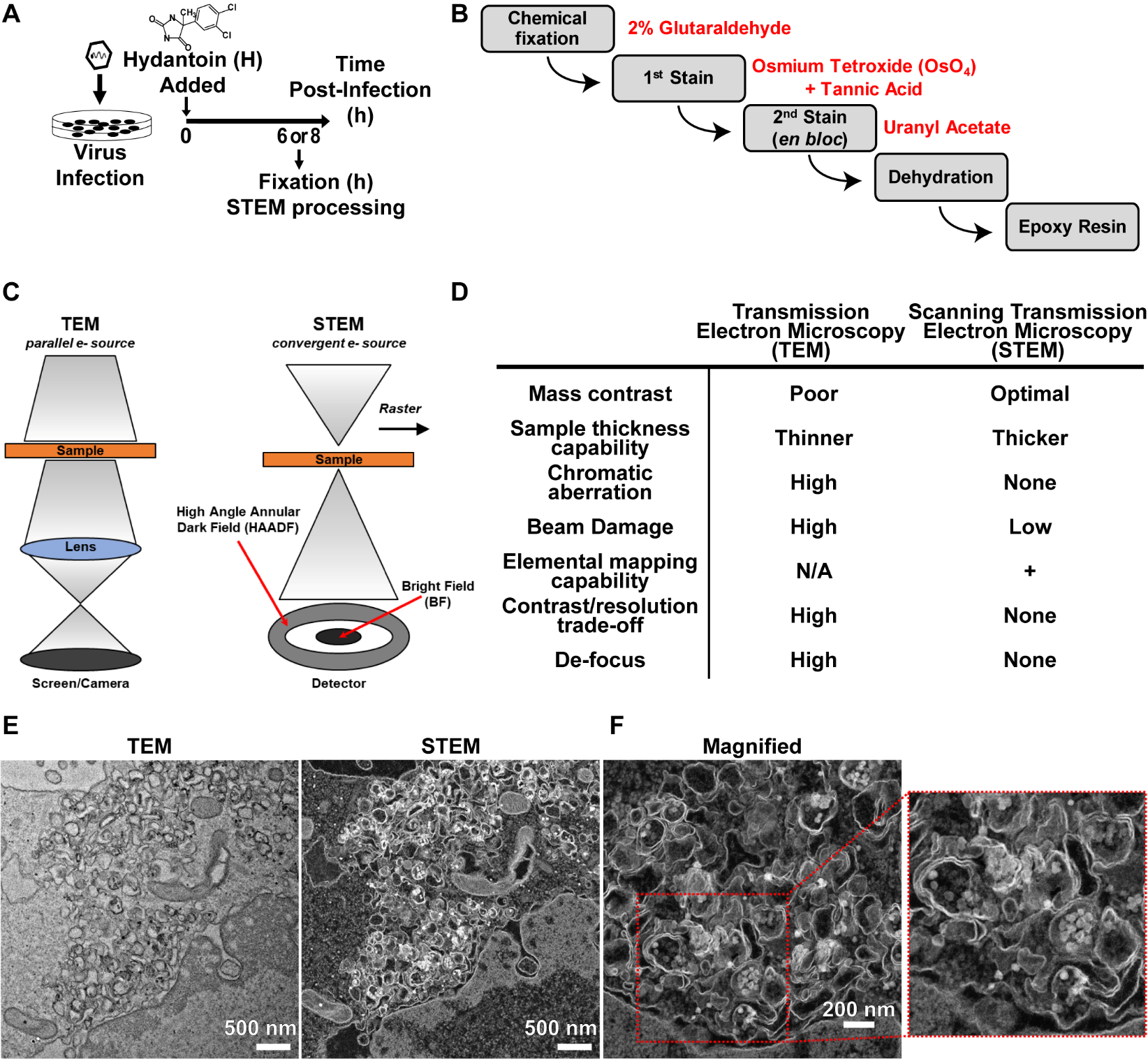
**

**Figure S2. An alternate imaging approach: Scanning transmission electron microscopy (STEM).**

**(A)** **Cell lysate preparation for STEM.** Infection of HeLa cell monolayers was carried out in the presence or absence of hydantoin. A cell suspension is then prepared using trypsin to release the monolayer at the stated time points 6 or 8- hours post-infection. Cells are gently pelleted, fixed, and processed as described in panel **(B)**. **(B)** **Cell microsection preparation for STEM.** Cell pellets were subjected to chemical fixation using 2% glutaraldehyde. An initial stain was performed using osmium tetroxide, followed by tannic acid treatment. A second *en bloc* stain was completed using uranyl acetate. Cell pellets were then dehydrated and embedded in an epoxy resin. Thin microsections were then collected and placed on a carbon-coated grid, where a third and final on-grid stain was performed. **(C) Schematic of TEM and STEM microscopy**. TEM is set up much like light microscopy but uses electrons and electromagnetic lenses instead of light. Briefly, the beam hits the sample, electrons are scattered, and the lens forms an image projected to the camera. STEM is entirely different. The beam is converged to a single point, then rastered across the sample, and a detector collects the resulting scattered electrons. In short, TEM contrast comes from unscattered electrons. In STEM, contrast comes from scattered electrons. **(D)** **Advantages and disadvantages of TEM and STEM imaging.** This table discusses the advantages and disadvantages of Transmission Electron Microscopy (TEM) and Scanning Transmission Electron Microscopy (STEM) to provide some perspective on the factors influencing the contrast gains obtained when imaging biological samples using STEM. In short, we enumerate several advantages of using STEM imaging in the ultrastructural analysis of biological samples, such as membrane derangements in infected cells. **(E) TEM and STEM imaging mode comparison.** HAADF-STEM (High Angle Annular Dark Field - Scanning Transmission Electron Microscopy) imaging of WT PV-infected HeLa cells. HeLa cells were infected with WT PV at an MOI of 10 and then fixed in glutaraldehyde 6 hours post-infection (hpi). Fixed samples were dehydrated, stained, embedded, and sectioned in thin micrographs for imaging as described in panels **(A)** and **(B)**. Images were collected using a Thermo Scientific Talos F200X G2 (S)TEM operated at 200 kV and a beam current of approximately 0.12 nA. The contrast is also reversed when compared to TEM, with the vacuum appearing dark. WT infection induces virus-containing double membranous vesicles and multi-vesicular amphisome-like vesicles with virions in the intra-luminal vesicles. Large outer vesicles with intra-luminal vesicles (100-300 nm diameter) contain ~30 nm particles inside. Double membrane vesicles are located at sites where vesicular-tubular clusters are observed in TEM mode. **(F)** **STEM imaging of WT PV-infected HeLa cells (magnified)**. In this magnified view, we look closely at observed structures in panel **(E)**. Large outer vesicles with intra-luminal vesicles (100-300 nm diameter) contain ~30 nm particles inside. Double membrane vesicles are located at sites where vesicular-tubular clusters are observed in TEM mode. 30 nm virus particles observed inside of intra-luminal vesicles. Close-up view of an intra-luminal vesicle that contains 30 nm particles.

**
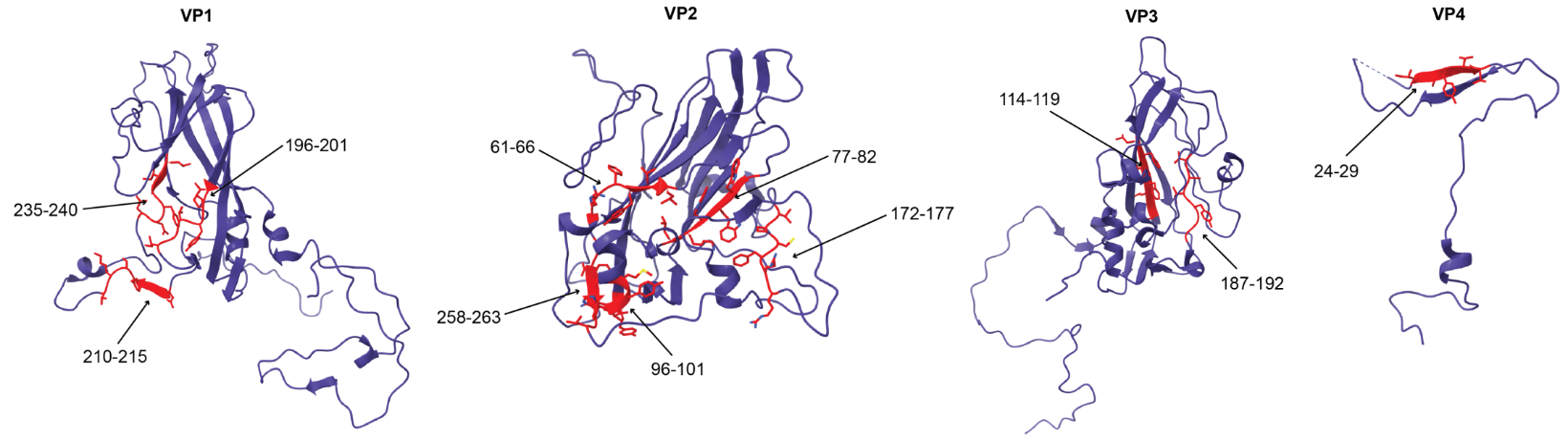
**

**Figure S3. LC3- and GABARAP-interacting regions in PV structural proteins**

LC3-interacting region (LIR) mediates LC3 binding with autophagy-associated factors and cargo. LIRs are characterized by a consensus motif (W/F/Y) (x) (x) (L/I/V). All PV protein products encode at least 1 LIR for a total of 33 across all PV proteins. Shown in violet are ribbon depictions of VP1, VP2, VP3, and VP4. The VP1 region encodes 3 LIRs, VP2 5 LIRs, VP3 2 LIRs VP4 1 LIR all shown in red.

**Movie S1. Bulk spread assay movie.** HeLa cells in suspension were stained using a membrane dye and infected with a green fluorescence PVeGFP**_pv_** reporter variant. MOI of 5-infected dyed cells (red) were washed and seeded on top of a naïve HeLa cell monolayer. Fluorescence is monitored over time to detect both primary and secondary infections. Primary infected cells were observed and depicted in yellow when green (eGFP expression) and red signal (cell dye) colocalized. Spread was detected when a secondary wave of PV green fluorescence signal (green only) originating from the newly infected monolayer of unstained cells was observed.

**Movie S2. Epifluorescence imaging of single-cell pairs movies.** Representative fluorescence movies of chambers harboring cell pairs in a single-cell spread assay. Cells in suspension infected with a reporter PV-unaG_pv_ virus variant (green). Infected cells were paired with stained uninfected cells (red) in isolated chambers of a multi-chamber microfluidics polyvinylidene fluoride (PVDF) device. In this study, this device was modified to harbor cell pairs. Fluorescence is monitored over time to detect an initial wave of infected cells expressing green fluorescence, yielding a yellow fluorescence overlay (see yellow cells). Spread was detected when a secondary wave of green fluorescence signal was observed in red-dyed cells, producing a colocalized yellow signal. Spread events were further extrapolated into no-spread, lytic spread, and non-lytic spread. **(A)** In lytic spread, the secondary infection signal arose after losing the primary cell green fluorescence (lysis). **(B)** In non-lytic spread, the secondary infection signal was detected while green fluorescence was still present in the primary infected cell. **(C)** In no spread, no secondary infection signal was detected after a primary cell green fluorescence signal.
